# Educating Early Adolescents for a Sustainable Future With Digital Civic Learning: Moral Self‐Concept as a Developmental Catalyst Linking Civic Competencies and Civic Purpose

**DOI:** 10.1111/desc.70203

**Published:** 2026-05-07

**Authors:** Saetbyul Kim, Tzu‐Jung Lin, Michael Glassman, Eric Anderman

**Affiliations:** ^1^ The University of Hong Kong Hong Kong China; ^2^ The Ohio State University Columbus Ohio USA

**Keywords:** civic competencies coding scheme, civic purpose, digital civic learning, moral self‐concept, sustainable development goals

## Abstract

Education for sustainable development (ESD) is increasingly recognized as a developmental imperative in a world facing ecological, social, and political challenges. While many approaches to ESD emphasize knowledge and competencies, emerging research suggests that sustainable action in youth requires the integration of cognitive, emotional, and identity‐based developmental processes. We propose that Digital Civic Learning (DCL) serves as a developmentally appropriate educational approach that can scaffold this process in early adolescence. Using a sample of 149 4th and 5th graders in Mid‐Western United States, we examined how civic competencies, namely civic knowledge, skills, and dispositions, relate to civic purpose and further investigated the mediating role of moral self‐concept. Our findings underscore the importance of nurturing moral self‐concept so that enhanced civic competencies translate into a form of civic purpose.

**Summary:**

- Conceptual innovation: It introduces Digital Civic Learning as a design‐based, developmentally aligned model that integrates civic learning with sustainable development goals, aiming for transformative impact in education.
- Methodological advancement: It develops and applies a novel civic competencies coding scheme tailored for a nuanced analysis of students' civic knowledge, skills, and dispositions.
- Theoretical contribution: It positions moral self‐concept as a developmental link connecting civic learning with a sustainability‐oriented civic purpose in early adolescence, a critical period for identity formation.
- Empirical rigor: It employs performance‐based assessments to evaluate students' civic competencies demonstrated in authentic digital classroom settings, emphasizing real‐world application of civic reasoning within sustainability education.

## 1 Introduction

Education for sustainable development (ESD) has been broadly defined as the process of equipping learners with the knowledge, competencies, values, and attitudes necessary to address complex and interdependent sustainability challenges (UNESCO 2020). Although traditionally framed as a curricular and pedagogical reform, we argue that ESD must also be understood from a developmental perspective. Early adolescence marks an important developmental period for several critical competencies, including those related to citizenship and social participation for cognitive, socio‐emotional, and moral growth (Dahl et al. 2018). Key characteristics of ESD include future‐oriented reasoning, critical reflection, and collective action (Redman and Wiek 2021). These characteristics align with developmental shifts occurring between the ages of 9 and 12, such as the growth of future oriented thinking and self‐representation (Nurmi 1987), social awareness (Damon and Hart 1982), critical argumentative thinking (Lin et al., 2019, 2022), prosocial moral reasoning (Spinrad and Eisenberg 2014), social perspective taking, and collective efficacy (Lu et al. 2023). Importantly, this is also a time when early adolescents begin integrating abstract moral values into their self‐concept, forming the foundation for long‐term identity development (Hardy and Carlo 2011). The purpose of this study is to identify factors that contribute to early adolescents’ sustainable civic development under a developmentally appropriate curriculum called digital civic learning (DCL).

Specifically, we first examine how fourth‐ and fifth‐grade US students’ civic knowledge, skills, and dispositions emerge as they engage in productive and meaningful classroom discourse in DCL—including individual reflective essays, self‐recorded videos, and online discussions. We further explore how these competencies inform students’ moral self‐concept and civic purpose, defined as the intent to improve society through civic actions. By employing a novel assessment approach that captures students’ real‐life civic competencies within their learning environments, the study offers an authentic portrayal of early adolescents’ civic development and highlights the catalyzing role of moral self‐concept in shaping civic purpose.

## 2 Literature Review

### 2.1 The Importance of Early Adolescents’ Civic Purpose in Sustainable Development

Acquisition of civic competencies is essential in preparing for a sustainable future; however, research increasingly suggests that such skills alone are not sufficient for fostering long‐term engagement in socio‐political contexts (e.g., Manganelli et al. 2014). Young people may know what to do to make productive change, and even how to do it, but whether they choose to act often depends on deeper motivational and identity‐related factors (Blasi 1983; Thomaes et al. 2023). Civic purpose refers to a sustained intention to contribute to the common good, involving three distinct aspects: civic intention, motivation, and actions (Malin et al. 2015). Individuals who are civically purposeful perceive civic participation as personally meaningful and important (civic intention dimension), particularly based on prosocial, self‐transcendent, and community‐based reasoning (civic motivation dimension), followed by corresponding relevant engagement in civic behaviors (civic action dimension). Therefore, civic purpose is a multidimensional developmental construct that reflects individuals’ progression toward mature citizenry. These pillars of civic purpose are aligned with the aims and goals of sustainable development, which seek to ensure dignified lives for all people—both now and in the future—while maintaining the sustainability of environmental, economic, and social systems (see United Nations 2015; United Nations Department of Economic and Social Affairs [UNDESA] 2025).

In order for adolescents to engage in sustainable behaviors, research suggests that they should construe societal good and sustainable development as aligned with their values and personal motives (Thomaes et al. 2023), which is captured in both the *intention* and *motivation* dimensions of civic purpose. When adolescents personally evaluate civic behaviors as not only societally but also personally meaningful, and when such perception is motivated by their desires to contribute to broader communities, they can readily engage in civically desirable actions for a sustained period. This is supported by research demonstrating that the integrated development of the three dimensions of civic purpose leads adolescents to maintain civic engagement during emerging adulthood, despite the general decline in civic behaviors after high school (Malin et al. 2017). Self‐transcendent civic motivation predicts stable, sustained participation in civic activities (Malin et al. 2015) and the alignment of personal values and targeted behaviors may prompt adolescents to engage in socially desirable behaviors (Yeager et al. 2018). Taken together, to induce sustained civic endeavors and engagement in young generations, it is necessary for adolescents to understand and approach societal, civic, environmental issues as societally and personally meaningful to their lives, which is well reflected in the construct of civic purpose.

Early adolescence is an auspicious developmental period for nurturing civic purpose, as early adolescents are developing more mature reasoning about the broader consequences of their actions (Casey et al. 2008), while also developing moral emotions such as guilt and empathy (Schalkwijk et al. 2016). Furthermore, early adolescents’ cognitive flexibility (Buttelmann and Karbach 2017), social perspective taking (Kim et al. 2024), and sense of justice (Bondü and Kleinfeldt 2021) are advancing, with a more complex, nuanced moral self increasingly shaped by social contexts (Sengsavang and Krettenauer 2015). With these developmental changes, early adolescents become increasingly capable of recursive and multicausal reasoning, enabling them to grasp feedback loops, unintended consequences, and systemic relationships (Davis and Sumara 2006; Piaget 1970). Though less frequently studied in children and early adolescents, evidence suggests early adolescence is a fertile time for initial existential questioning and the integration of values into the self‐concept, especially when scaffolded by culturally sensitive educational contexts (Damon 2009; King and Boyatzis 2004). Such developmental momentum in early adolescence is often neglected due to systematic constraints and adults’ skepticism about young students’ capacities to make productive change (Lundy 2007).

With that, well‐designed ESD needs to move beyond just teaching “about” sustainability. Meaningful educational processes should actively support the internalization of sustainability values into the self—helping students see themselves not only as learners, but also as responsible stewards and contributors to an interconnected world. It is essential to design and formulate learning environments that move beyond teaching “what” and “how” to also shaping “who,” supporting early adolescents in seeing themselves as agents of sustainability.

### 2.2 Digital Civic Learning as a Developmentally Aligned Pathway to Sustainability‐Oriented Civic Purpose

#### 2.2.1 DCL as Education for Sustainable Development

Digital civic learning (DCL) is an instructional approach designed to foster civic competencies, moral self‐concept, and a sustainability‐oriented civic purpose in early adolescents through immersive, discourse‐rich learning experiences. The DCL curriculum was iteratively developed over 2 years, based on student and teacher feedback. It integrates two instructional features: collaborative social reasoning (Lin et al. 2022; adapted from collaborative reasoning, Anderson et al. 2001) and immersive learning (Kuznetcova et al. 2025). Grounded in educational and developmental theories (Anderman et al. 2024; Glassman 2001; Vygotsky 1987) and guided by psychological principles such as agency, We‐ness, and empathy (Kim et al. 2025), this approach engages early adolescents in respectful, collaborative reasoning about socially and morally complex issues and places them in immersive scenarios that foster historical understanding and collective problem‐solving. DCL reading materials were selected so that they are aligned with early adolescents’ cognitive development (Piaget 1962), and linguistic/vocabulary skills (Hsieh et al. 2021; Lin e al. 2015). Using heterogenous grouping, students experience diverse perspectives in group settings that promote their social perspective taking and enrich their social experiences (Kim et al. 2020; 2024). DCL additionally utilizes interactive low‐tech curricular materials that do not place technical burdens on students or teachers (Kuznetcova et al. 2025), while engaging students into personally relevant activities (Anderman et al. 2024) and argumentation (Park et al. 2025) through online platforms (Cha et al. 2025). Implemented through four units, DCL presents learners with civic oriented problems that mirror real world challenges, such as land rights, housing justice, environmental degradation, and profit maximization, tapping into three pillars of sustainability: economic (profit), social (people), and environmental (planet) (United Nations 2015). The problems are intentionally designed to evoke moral tension, invite multiple perspectives, and promote systems thinking—central goals of education for sustainable development (ESD).

Each DCL unit unfolds in three interconnected phases that resonate with core ESD pedagogical principles—critical inquiry, participatory learning, and values reflection (see Parry and Metzger 2023). Specifically, During DCL, students learn social issues for sustainability not only by exploring the historical and social backgrounds of a problem, but also by participating in developing solutions based on increased understanding of the issue. While participating in small‐group projects in face‐to‐face and asynchronous online formats (e.g., self‐recording video essays, Web 2.0 technologies, online discussion boards), students adopt stakeholder roles—such as affected families, community members, or government representatives—as they search for workable solutions to ill‐structured problems at hand (e.g., What would Ms. Haaland^1^ tell other government leaders and why?). These simulations require collaborative reasoning and ethical deliberation in response to problems that reflect the complexities and uncertainties of sustainability dilemmas, challenging students to reconcile competing interests and consider long‐term consequences.

Through Collaborative Social Reasoning (CSR) processes, students are given the space to co‐construct arguments, test the validity of their assumptions, and engage with multiple perspectives (Kim et al. 2024; Kraatz et al. 2020; Lin et al. 2019; 2022; Nagpal et al. 2025; Wen et al. 2023). This dialogic process models participatory democracy and enhances students’ critical inquiry, both of which are central to ESD's aim of preparing individuals for responsible civic and environmental decision‐making. By becoming a participant in the decision‐making process of a virtual community with varied stakeholders, students are asked to consider multiple perspectives as lived experiences, joining in the “vivencia” of their adopted community (Glassman and Erdem 2014, 213). Such experiences of psychological engagement support the development of empathy and moral reflection, which are critical for the values‐based orientation of ESD, by effectively helping students internalize the values of morality (e.g., honesty, sympathy) and perceive societal concerns as important at both personal and social levels. Recurring discussion and essay prompts embedded in the curriculum further encourage students to reflect on what matters to them personally as they critically interrogate their own value systems.

One of the most important aspects of DCL, when considering ESD in the 21st century, is its digital‐based, collaborative curriculum, which fosters a sense of shared and collective responsibility both online and offline. Such DCL designs that incorporate use of digital tools and collaborative participation in digital learning space naturally provide students with opportunities to grow with digital citizenship. Digital citizenship in the increasingly networked online space creates a means for sustainable and sharable identity among individuals (Castell 1996). Every point in a vast interconnected electronic network has the potential to act as both consumer and producer of new information in ways that can alter online discourse, especially concerning problem‐solving (Selwyn 2009). DCL strategically responds to the penetration and pervasiveness of Internet technologies into everyday activities in the 21st century. Specifically, students are positioned as agentic consumers and producers of civic discourse, tapping into the required abilities of digital citizenship, recognizing and taking responsibility for emerging non‐hierarchical, non‐linear information ecologies in the information networks (Choi et al. 2017).

### 2.3 Moral Self‐Concept Linking Civic Competencies and Civic Purpose During DCL

#### 2.3.1 Civic Competencies

Civic competencies, a set of abilities essential for active citizenship (Hoskins et al. 2015), are defined and conceptualized in diverse ways (Sherrod 2015; Torney‐Purta et al. 2015). Nonetheless, there is agreement among researchers that civic competencies are multidimensional, encompassing skills, knowledge, attitudes, and dispositions (e.g., Metzger et al. 2018; Wray‐Lake et al. 2017). This study specifically refers to the National American Educational Progress framework (NAEP 2018, as cited in Lee et al. 2021; National Center for Education Statistics n.d.) that broadly approaches civic competencies as three dimensions: civic knowledge, skills, and dispositions. Accordingly, we operationalize the concept of civic competencies as a set of abilities that citizens can demonstrate and perform, including their knowledge, skills, and dispositions in authentic contexts. We approach civic competencies specifically “in context,” suggesting a need to capture individuals’ performance not as a self‐reported evaluations or performance as a form of tests, but rather as competencies demonstrated in authentic settings, either individually or collectively.

Civic knowledge (e.g., knowing current events, government systems) and skills (e.g., writing an opinion letter) predict one's future intention for civic actions (Cohen and Chaffee 2013), suggesting such skill sets serve as meaningful antecedents for one's citizen life. Civic skills, such as describing, identifying, and elaborating on ideas (Zorwick and Wade 2016), co‐constructing knowledge with others (Akar 2016), as well as listening to others (Kirlin 2003), are essential capacities for competent citizenry (Rutten and Soetaert 2013).

Some scholars claim that competencies alone are not sufficient for sustaining long‐term civic engagement in future activities (outside the formal classroom) (Ballard and Ozer 2016; Rimanoczy and Llamazares 2021). To move from skills to enduring purpose, learners need to experience integration of those competencies into their “self‐driven” activities—a process grounded in identity development and moral motivation. The ESD literature describes the process as “being” as opposed to “doing,” “thinking,” or “knowing.” (see Cripps and Smith 2024; Fairfield 2018). With that, we posit that moral self‐concept, a psychological construct representing one's perceived importance of moral values to the self, may act as a mechanism translating one's civic competencies into civic purpose. Students who develop competencies during the DCL may only translate these into action if they also see such engagement as part of “who they are.” In this view, moral self‐concept acts as a developmental bridge between what students can do (competencies) and what they choose to do in pursuit of sustainability (purpose).

#### 2.3.2 Moral Self‐Concept

Morality is a complex psychological construct that can be defined in various ways, involving multilayered, multifaceted influences on its development. Aside from individuals’ morally relevant tendencies (e.g., Eisenberg et al. 1998), social contexts and environmental influences such as socialization with peers, access to media, involvement in community activities, cultural norms, and prompted reasoning on moral dilemmas all contribute to adolescents’ moral development (e.g., Hart and Carlo 2005; Shweder et al. 1987; Thompson 1971). In line with this, we position morality as individuals’ desirable virtues that are central in human flourishing toward creating the best possible world (see William 1995), yet its development is largely shaped by contextual, sociocultural, and environmental factors (Hart and Carlo 2005), rather than advancing effortlessly as one's cognitive capacity grows.

We also adopt the definition of moral self‐concept as the extent to which individuals internalize and identify with their purported moral “values” (e.g., justice, honesty, compassion) (Gibbs 2019; Patrick et al. 2018). Not everyone who understands what should be done or what constitutes best practices for society necessarily act in accordance with these beliefs. This gap is often ascribed to the degree to which moral values are central to one's identity. When such values are perceived as core to the self, individuals are more likely to engage as responsible citizens concerned with the well‐being of others and the sustainability of society. For instance, a classic study by Hart and Fegley (1995) found that care exemplar adolescents, nominated by community members, did not necessarily demonstrate higher moral reasoning than the comparison group. Rather, they identified themselves using more moral, positive, and caring descriptors than others, suggesting that higher centrality of moral values in oneself (i.e., moral self‐concept) leads to higher commitment as citizens (i.e., civic purpose) during adolescence. A study by Porter (2013) on older adolescents also showed that adolescents’ moral self‐concept, when controlling for political self‐concept, explains unique variance in civic involvement: higher moral self‐concept predicts higher civic commitment. Thus, many scholars argue that moral self‐concept functions as a motivational driver for moral behavior (Krettenauer and Hertz 2015). Given that many forms of civic engagement are viewed as inherently prosocial and moral in nature, moral self‐concept may serve as a significant predictor of the development of civic purpose (Metzger et al. 2018).

ESD frameworks emphasize cognitive and social‐emotional competencies as critical for informed and responsible action (UNESCO 2020; Redman and Wiek 2021). However, without a sense of personal relevance or moral investment steering students’ activities, even well‐developed skills may not easily translate into sustained civic or sustainability behaviors (Hardy and Carlo 2011; Malin et al. 2017). This disconnect underscores the critical role of moral self‐concept—a bridge between one's civic and moral motivations and their corresponding actions (Blasi 1983; Colby and Damon 1992).

#### 2.3.3 Civic Purpose

We posit that civic purpose, one's future oriented and prosocial intention to contribute to the common good by participating in civic activities, is a primary outcome of DCL. Within the framework of ESD, civic purpose moves beyond technical “how to” knowledge to address the “why” of engagement, framing students as social agents committed to societal well‐being.

Previous findings highlight the importance of valid social inputs, optimal surroundings, and well‐designed educational programs in fostering civic purpose in adolescents. For instance, Burrow (2015) asserted that not every adolescent is surrounded by meaningful opportunities for civic engagement that could lead to the development of civic purpose; hence it becomes important to create partnerships with social figures or organizations to promote civic purpose, which can speak to the crucial role of teachers as facilitators in classrooms. Han et al. (2019) also found that adolescents’ moral identity significantly predicted formation and maintenance of political purpose, a more specific and narrowed form of civic purpose bounded in por litical activities. Though their sample was high schoolers, the finding still suggests a possible role of moral self in informing civic purpose development in early adolescents. Quinn and Bauml (2018), investigated  how 6th – 9th grade adolescents’ insights they gained during a five‐day civics camp relate to their civic profiles, suggesting linkages between educational experiences and civic purpose. Bauml et al. (2023) later identified three core components of a civic learning approach that effectively foster civic purpose: developmentally appropriate activities, adult guides, and action‐oriented civic curricula.

#### 2.3.4 The Current Study

The current study investigated the relationships among three constructs central to developmentally informed ESD: civic competencies, moral self‐concept, and civic purpose in DCL context. Our aim was to empirically examine whether and how moral self‐concept mediates the association between early adolescents’ civic competencies and their civic purpose for a sustainable future. The DCL curriculum contains four 10‐day units, namely Geography, Citizenship, History, and Economics. For this study, data were collected over two units: Unit 3 (History) and Unit 4 (Economics). Units 1 (Geography) and 2 (Citizenship) were not included in this study because they represented a transition phase during which students and teachers were still adjusting or adapting to the new curriculum. It was not until Unit 3 that classroom interactions and behaviors fully adhered to DCL norms, remaining stable through Unit 4. Two research questions were examined: (1) Do students who show higher levels of civic competencies during their participation in Digital Civic Learning demonstrate greater civic purpose? (2) Does students’ moral self‐concept mediate the association between their civic competencies (knowledge, skills, and dispositions, respectively) and civic purpose?

## 3 Method

### 3.1 Study Context

#### 3.1.1 Sample

Participants were 149 4th (50.3%) and 5th (49.7%) graders (*M*_age_ = 9.92, *SD* = 0.71) and eight teachers (four in the 4th grade, four in the 5th grade) from six schools in the two districts in the Midwestern areas of United States. Students and parents were informed about the study objectives and procedures. Those students who assented to participate and whose parents also permitted participation served as participants. All procedures and assessments had been approved by the Institutional Review Board. In this sample, 61.3% were White, 16.7% Black, 5.6% Hispanic, 12.7% Asian, and 3.7% multi‐racial and others. About 47.6% of students were female, and 9.3% students were English learners. According to district‐data, about 14.1% of students received free and reduced lunch.

#### 3.1.2 Teacher Workshop

Teachers participated in a 4‐week online professional development workshop to prepare for the implementation of the Digital Civic Learning (DCL) curriculum in their social studies classrooms. The workshop introduced teachers to the study's goals and timeline, the key principles of DCL activities and instructional approaches, the assessment plan, and an overview of the curriculum. Emphasis was placed on instructional strategies that promote productive dialogue, collaboration, and culturally responsive practices when addressing complex social issues relevant to students’ lived experiences. The workshop included weekly online or in‐person meetings and ongoing dialogue through a dedicated social media platform. Upon completion, teachers were asked to rate the usefulness of the workshop. The average rating was 4.48 out of 5 (*SD* = 0.53), indicating that participants found the workshop helpful in preparing for DCL implementation.

#### 3.1.3 Procedure

Each unit consisted of 10 school days’ lessons, and on average, it took about 2–3 weeks for each teacher to complete one unit, mainly because some teachers spent more time debriefing the lesson purpose, allowed additional time for collaborative discussion among students, and oftentimes, there were days with no DCL lessons due to the school events or weather. Throughout the DCL, students were immersed into cultural and historical contexts to learn about civic topics and indirectly experience situations of others through whole‐group and small group‐based audio listening, video watching, and activities (e.g., a time warp, identity tree, and group shopping). Lessons started with the review of the previous day's highlights to help students experience sequential, connected, and coherent learning throughout the curriculum. Students spent 2 days (Day 3, 9) discussing suggested prompts (e.g., *“What advice would you give to the people in charge of deciding whether a pipeline should be built? Why do you think this advice would be helpful?”*) in both small and whole group settings, involving face‐to‐face and online discussions. Discussion prompts were derived from assigned readings, videos they watched, and class activities. Students were asked to express their thoughts and feelings in the form of self‐recorded videos, reflective essays, and interactive online discussion threads embedded in the Learning Management System (LMS).

Self‐reported survey assessments (moral self‐concept, civic purpose) were administered twice before Unit 3 and after Unit 4. Qualitative data were collected while students participated in the DCL curriculum, using multiple sources representing student participation, namely group discussion threads, self‐recorded videos, and individual essays submitted on Day 9 of Unit 3 and 4. Civic competency indices were calculated based on the qualitative data derived from such simulated public deliberation, community storytelling, and ethical dilemma discussions related to environmental and social justice (see Section null below).

Using regression analyses, we examined whether civic competencies predicted civic purpose. Using a mediation analysis, we tested whether civic competencies (i.e., knowledge, skills, dispositions) predicted civic purpose and whether this relationship is mediated by moral self‐concept.

### 3.2 Measures

#### 3.2.1 Moral Self‐Concept

Moral self‐concept was assessed using the Moral Self‐Relevance Measure (MSR; Patrick and Gibbs 2012). Participants were presented with eight moral attributes (e.g., *considerate*, *honest*) and eight non‐moral attributes (e.g., *funny*, *outgoing*). To enhance clarity, each item was accompanied by two synonyms (e.g., “How important is it to you that you are generous or giving?”). Participants rated the importance of each trait on a 5‐point Likert scale ranging from 0 (*not important*) to 4 (*extremely important*). Non‐moral items were included to obscure the primary focus of the measure. The MSR has demonstrated strong internal consistency for the moral subscale (*α* = 0.83–0.90; Patrick and Gibbs 2012, 2016; Patrick et al. 2018). In the current study, internal consistency for the moral subscale was satisfactory at both before Unit3 and after Unit4 (T1: *α* = 0.813; T2: *α* = 0.852).

#### 3.2.2 Civic Purpose

Civic purpose was assessed with three subscales (intention, motivation, action), informed by Malin et al. (2017). The *civic intention* subscale assessed the extent to which students perceived civic activities as important and meaningful in their lives, using an adapted version of Malin et al.’s (2017) scale. This subscale has demonstrated good reliability (*α* = 0.77–0.81; Malin et al. 2015, 2017). Students rated the importance and meaningfulness of five civic activities (e.g., “making a difference through volunteering”) on a 5‐point Likert scale ranging from *not at all meaningful* to *extremely meaningful*. Minor wording adjustments were made for developmental appropriateness while preserving original meanings (e.g., “being involved in politics” was rephrased as “participating in political activities like voting, campaigns, or protests”) (see full items in Kim, 2023). Internal consistency in the current study was acceptable (T1: *α* = 0.768; T2: *α* = 0.807).

The *civic motivation* subscale assessed students’ underlying reasons for civic participation, based on Malin et al. (2017), which was developed from themes identified in Ballard's (2015) qualitative research. The original 12‐item scale included both self‐oriented (e.g., “to meet school's requirement”) and beyond‐the‐self (e.g., “to give back what I have been given”) motivations. In this study, rather than using a ranking method, items were presented on a 5‐point Likert scale to generate average scores. To reduce participant fatigue, the scale was shortened to eight items—four for each motivation type. Only the beyond‐the‐self subscale was used, aligning with the conceptual focus on civic purpose. Internal consistency was *α* = 0.676 (T1) and *α* = 0.753 (T2).

The *civic action* subscale captured students’ engagement in civic behaviors using a modified version of the Youth Inventory of Involvement measure (Pancer et al. 2007), previously validated by Malin et al. (2015) to include three reliable factors: political/leadership activities, expressive activities, and community service (*α* = 0.70–0.83). The original 15‐item scale was shortened to six items, with simplified wording appropriate for fourth and fifth graders (Kim, 2023). For instance, “giving money to a cause” was revised as “donating money or other things (e.g., food, clothes) to people who are in need.” Items were rated on a 5‐point Likert scale. Internal consistency for this subscale was *α* = 0.716 (T1) and *α* = 0.675 (T2).

The overall internal consistency of civic purpose, combining three subscales, was satisfactory (*α* = 0.808 for T1, *α* = 0.851 for T2).

#### 3.2.3 Civic Competencies (Knowledge, Skills, Dispositions) in Digital Learning

Students’ *civic knowledge, skills, and dispositions* arising from immersive learning and civic reasoning during the DCL curriculum were assessed using three primary sources of data collected via students’ participation in multimodal activities: (1) weekly online discussion board posts submitted at the end of each unit; (2) individual online argumentative essays submitted at the end of each unit; and (3) video reflections recorded at the end of each unit using a self‐recording software, Flip. These data sources were selected because they provide valid insights into students’ civic understanding and development as summative assessments for each unit. Civic competencies, as assessed in this study, represent students’ engagement and expression in various communicative forms (e.g., written forms, verbal forms) “in” digital spaces and “using” digital tools (e.g., online learning platforms, digital software).

To analyze these data, we developed a civic competencies coding scheme based on the Civics Assessment Framework suggested by the National Assessment of Educational Progress (NAEP 2018, as cited in Lee et al. 2021). This framework was chosen for its comprehensive coverage of essential civic competencies identified in the civic development literature, including civic knowledge, values, and skills (Galston 2007; Hamilton and Kaufman 2022; Metzger et al. 2018). It encompasses both cognitive and affective dimensions of civic reasoning, aligning with the goals of DCL to foster holistic civic development. To further develop a coding scheme tailored to capture students’ emerging civic competencies, while following the NAEP civics framework (i.e., a deductive approach), the primary coder used 25% of student‐generated responses to discussion/essay prompts (discussion board posts, self‐recording video essays, individual reflective essays) from Units 1 and 2 (pilot data not used for the study) to identify context‐specific civic competencies that were relevant to the characteristics of DCL (i.e., an inductive approach). Using both deductive and inductive approaches, the primary and secondary coders then conducted content analysis (Kondracki et al. 2002) to additionally identify themes and iteratively develop and finzlie the coding scheme, drawing on discourse data from Units 3 and 4 (data used for the study).

The qualitative data (video essays, discussion board posts, reflective essays) were segmented into sentences as the unit of analysis. Each sentence was coded with respect to three dimensions of civic competencies (civic knowledge, skills, dispositions.) For example, if a sentence involved civic knowledge, a specific subcode of civic knowledge was assigned, such as “civil society.” The same sentence was simultaneously coded for civic skills (e.g., elaborating) and dispositions (e.g., prosocial). If a sentence did not involve any respective civic competencies, it was coded as NA (nonapplicable). It was possible that a sentence demonstrated one dimension of civic competencies but not the others. The number of sentences with an assigned subcode was calculated for each dimension for each student, respecting their levels of civic knowledge, skills, and dispositions. It was also possible that a sentence involved more than one codable subcode in one dimension. For instance, a sentence “*I like the idea but I also would like to know more*” could be coded as either “praising” or “asking for clarification” for civic skills dimension. The coder(s) evaluated which aspect was closer to the main purpose of the comment made by the interlocutor, especially when considering how the sentence connected to previous and later comments for contextual validity. In this example, given that the positive comment was made to clarify the student's further request, the coder(s) assigned “asking for clarification” rather than “praise.” Hence, all sentences were assigned only one sub‐code under each dimension of civic competencies. Decisions about the most dominant subcode for sentences that potentially involved more than one codable subcode had minimal influence on the study's findings, because the subsequent analysesfocused mainly on the number of sentence units coded as valid civic competencies (i.e., sentences not coded as NA), which did not require differentiation among subcodes.

To assess inter‐rater reliability, the primary coder coded the full dataset, while a second coder independently coded 20% of each data source (i.e., 20% of Flip responses for Units 3 and 4, 20% of online discussion board posts, and 20% of individual written essays). Inter‐coder reliability was evaluated using Cohen's Kappa and demonstrated satisfactory agreement across all civic domains: civic knowledge (*κ* = 0.826), skills (*κ* = 0.873), and dispositions (*κ* = 0.996). Students’ levels of civic knowledge, skills, and dispositions were then determined by counting the number of applicable subcodes that appeared in their responses, based on the finalized codes established by the primary coder.

#### 3.2.4 Civic Knowledge

*Civic knowledge* dimension concerns “what” students “know” about the social world, including structures, systems, histories, and the interrelations among them. The definitions and examples are drawn from students’ participation in DCL activities (see Table 1). In this study, civic knowledge specifically encompassed content knowledge related to the history of Native Americans and economic knowledge associated with food insecurity—reflecting the thematic focus of the two curricular units on History and Economics. The civic knowledge dimension yielded six different subcodes: political and government system, civil society, social and historical events, social issues, economics, and international affairs. The sentences without civic knowledge were assigned NA.

**TABLE 1:** Knowledge dimension of civic competencies coding scheme (Kim 2023).

Dimension 1: Civic knowledge	
Subcodes	Definition and examples
Political and government system	Knowledge and understanding of formal government institutions. This includes government functions, types of government (democracy, dictatorship, monarchy, etc.), governmental organizations (e.g., branches of government, local, state, and federal government) and processes (e.g., how to pass a bill), rules and laws, including treaties, acts, regulations, citizenship rights, etc.
	e.g., *I also think the* ***government*** *is important because they need to make sure the people in their community have food security*.
	e.g., ***The government is in control of*** *businesses and so they need to make it so businesses are selling fresh food, otherwise families like Jeannie's will have a person sick*.
	e.g., *A* ***treaty*** *can be used by two or more groups of people, to solve a problem or to agree on something*.
Civil society	Knowledge and understanding of non‐governmental organizations including political (e.g., advocacy organizations), economic (e.g., trade associations, unions), social (e.g., community centers), cultural (e.g., museums), communications (e.g., media), and philanthropic (e.g., charities) organizations; how these institutions collaborate with and influence government; and how these institutions address social issues.
	e.g., *So then Cheryl and others who are doing this* ***community garden*** *can send it to others in need of the food and the food can be sent in boxes without the food rotting before it gets there*.
	e.g., *I would try to open a* ***business*** *and go for it, so I could help the community*.
	e.g., *On prompt 2, you could add the fact that* ***grocery stores*** *can also help the community by offering more than just food*.
Social and historical events	Knowledge and understanding of historical and social events including underlying causes, effects, impact, and relevance.
	e.g., *By approving this pipeline the government started a disagreement with the* ***Standing Rock Sioux tribe*** *because the pipeline cut under their water supply and through their cultural grounds*.
	e.g., *Eventually*, ***DAPL*** *got approved by the government, without the Sioux tribe getting talked to*.
	e.g., *In the* ***Indian removal act*** *, the seminoles wouldńt have enough space to get water and plant a lot of crops*.
Social issues	Knowledge and understanding of social issues that the government and civil society seek to address.
	e.g., *That is a lot of people that need help with* ***food security*** *and will get really sick and maybe even die without help*.
	e.g., *I disagree with prompt 2 because* ***a lot of people don't have jobs*** *and need them to get food for their families so if 12,000 jobs were presented, it would be amazing for many people that they now have a chance of getting a job*.
	e.g., *I'll explain that the pipeline is good because it will help manufacturers like transport oil and manufacture oil a lot and bad because* ***it will ruin the environment if it leaks***.
Economics	Knowledge and understanding of economic concepts such as supply and demand, scarcity, profit, saving, costs.
	e.g., *They will have to decide on whether to* ***spend money*** *on food or on medical help*.
	e.g., *It is also important because the* ***amount of money people would spend determines how much profit you get*** *and how much you can pay your employees*.
	e.g., ***Many neighborhoods do not have enough grocery stores*** *but grocery stores do not open in these areas because* ***they think they will not make enough profit to stay open***.
International affairs	Knowledge and understanding of how countries relate to and interact with each other, such as globalization, global conflicts, movement of peoples, international organizations, and international agreements.
	e.g., *As an example, if* ***three countries*** *agreed to give each other the resources they did not have*.
	e.g., *Another reason people think that the pipeline would be helpful, is because it would help the US become* ***less dependent on other countries*** *for oil*.
	e.g., *For example*, ***if you used to live in China and you moved to America*,** *you would probably still want to eat some cultural foods so that you can keep following certain traditions that you had in China*.
N/A	Does not involve any identified civic knowledge.
	e.g., *I also think that if the other side is not okay with it you can't do it unless you think it will cause a really big problem*.
	e.g., *And if you would do that, then it's basically just tearing up what's theirs*.
	e.g., *I had the mostly same ideas for my Flipgrid*.

These codes under civic knowledge are not only civically relevant but also deeply connected to key issues of sustainable development goals (SDG), such as reducing inequality (SDG 10), ensuring food security (SDG 2), and promoting inclusive societies (SDG 16). Understanding the historical marginalization of Indigenous communities and the structural causes of food insecurity helps students recognize how social, economic, and political systems contribute to injustice and unsustainability.

#### 3.2.5 Civic Skills

*Civic skills* are understood as “applying civic knowledge to good effect” (NCES n.d.). Civic skills are typically categorized into two broad domains: *intellectual skills* and *participatory skills*. According to the NAEP framework, *intellectual skills* include the ability to (a) identify and describe civic concepts, (b) explain and analyze ideas, and (c) evaluate, adopt, and defend positions—skills that reflect cognitive engagement and reasoning. In contrast, *participatory skills* pertain to interpersonal and social competencies, such as (a) interacting (e.g., cooperating with others), (b) monitoring (e.g., tracking civic or political developments), and (c) influencing (e.g., voting in a student organization). In this study, we focused on students’ *intellectual skills* and the *interacting* component of *participatory skills*, as these are most observable within a classroom setting (see Table 2). Civic skills dimension yielded a total of 10 subcodes aside from NA (nonapplicable): explaining/elaborating, positioning, justifying, hypothetical reasoning, causal reasoning, referencing, praising, expanding, asking for clarification, and simple dis/agreement.

**TABLE 2:** Skills dimension of civic competencies coding scheme (Kim 2023).

Dimension 2: Civic skills	
Subcodes	Definition and examples
Explaining/Elaborating	Providing personally interpreted descriptions o something; being able to make connections between different concepts or principles to demonstrate one's understanding.
	e.g., ***For example*,** *make the high‐tech lunch system not cost money or have it cost money but about 5 cents every meal*.
	e.g., *but* ***what I mean by that*** *is make the high‐tech lunch system fairer*.
	e.g., *if you break a peace treaty* ***that means*** *you will make enemies*.
Positioning	Being able to decide on one's position and clearly state one's thoughts.
	e.g., ***In my opinion*** *the school* ***should*** *keep the high‐tech lunch system*.
	e.g., ***The Government should*** *ask the Native Americans if they can change the treaty or break it*.
	e.g., *Sometimes*, ***it is okay to break a treaty*** *if you have talked to the people you are making the treaty with*.
Justifying	Connecting and providing relevant examples, evidence, and facts to support one's position.
	e.g., *I would leave some things behind* ***because*** *I would only need enough to support me during the pandemic*.
	e.g., *I think this* ***because*** *the government is in charge of the entire state so it is their job to protect it*.
	e.g., ***One reason*** *they are responsible for this is* ***because*** *just like the grocery store owners they are expected to have fresh food, and enough of it!*
Hypothetical reasoning	Reasoning about alternative situations and considering the flipped side of a standpoint.
	e.g., ***What if*** *you want something different other than food that you are getting every single day?*
	e.g., ***Without*** *the government's permission*, ***many poor people would*** *not be able to receive the food they need*.
	e.g., ***If*** *they are not stocked well enough then some people can get food*, ***whereas others*** *who may need it more have no access to free food*.
Causal reasoning	Reasoning about corresponding actions in consideration of the results, outcomes, and consequences held on others and communities; being conscious of the effects of a phenomenon and its causes.
	e.g., *I believe that* ***this could cause*** *bad feelings revolving around you*.
	e.g., *I would have to keep some of it to myself* ***so that I can*** *help the community and my family/fam*.
	e.g., *And the animals that eat the herbivores* ***might die from*** *eating the herbivores, and so on*.
Referencing	Referencing existing knowledge, information, or source; being able to expand and corroborate one's standpoint by mentioning relevant sources.
	e.g., ***A saying that goes along with*** *this is*, “*You got go slow so you can go fast later*.”
	e.g., ***Remember the example*** *of that man who ran such a huge grocery store in a food desert?*
	e.g., *Because it's like the saying*, ***I scratch your back, you scratch mine*,** *like a good version of* ***karma*** *if you help them and then you get help*.
Praising	Showing a positive reaction and offering encouragement to others.
	e.g., ***I like when you elaborated*** *by some fact you told you did not just say the fact and be done you elaborated on the fact that you told*. ***Good job!***
	e.g., ***I loved how you explained*** *what you would do in your opinion*.
	e.g., ***That was my favorite part!***
Expanding	Being able to make connections between others’ viewpoints or reasoning and one's own, contributing to the expansion of ideas (e.g., showing either agreement or disagreement to peers’ opinions with added thoughts).
	e.g., ***I also very much agree with the reason*** *to be nice if another student's feeling bad or having a bad day, but I think we should always be extra nice to everybody always, not just when they are feeling bad*.
	e.g., ***I agree with you if*** *the water is not clean people will die of thirst and they will get sick*.
	e.g., *I* ***respectfully disagree with you*** *because if we don't like change some holidays some people might get mad and most of the cultures don't make any sense to me*.
Asking for clarification	Being able to identify what they do not understand, acknowledge that they need more input or clarification, and ask others to elaborate or provide further information.
	e.g., *I think that* ***you could elaborate more on*** *both Prompts and add more reasoning*.
	e.g., *It would have been nice* ***if you said more reasonings*** *for your part one and two*.
	e.g., *But I think* ***you should have added more*** *pros and cons*.
Simple dis/agreement	Showing agreement or disagreement that does not seem to to expand the original ideas.
	e.g., ***Exactly!***
	e.g., ***I agree***.
	e.g., ***I don't think so***.
N/A	Does not involve any identified civic skills.
	e.g., *sry: (*
	e.g., *So thank you bye*.
	e.g., *That's it for day everyone*.

*Civic skills* align closely with the competencies emphasized in ESD and Sustainable Development Goal (SDG) 4.7, which highlight the importance of equipping learners with the ability to think critically, engage in dialogue, collaborate across differences, and take informed action toward sustainable futures. Intellectual and participatory civic skills enable young people to understand and address complex, interconnected global challenges such as climate justice and inequality, not merely as issues of knowledge, but as collective responsibilities. In this way, civic skills support the development of active, informed, and responsible citizens who can contribute to building a sustainable society.

#### 3.2.6 Civic Dispositions

*Civic dispositions* refer to the tendencies or orientations—such as critical consciousness, empathy, respect, and responsibility—that are fundamental to sustaining societal values and promoting the common good. The newly developed civic competencies coding scheme identified seven dispositions (see Table 3): empathy (empathetic), perspective taking (considerate), self‐transcendence (prosocial), social capital (open‐minded), respect for human dignity (respectful), critical consciousness (critical), and responsibility (responsible). Civic disposition codes were used to identify the underlying orientations that motivated and shaped students’ reasoning and discourse during their participation in DCL. These civic dispositions are also central to the goals of sustainable development, as outlined in SDG 4.7, which calls for education that promotes the knowledge, skills, values, and attitudes needed to live sustainably, promote human rights, gender equality, and a culture of peace and non‐violence. Dispositions such as empathy and critical consciousness empower students to recognize injustice, engage with diverse perspectives, and act in ways that support inclusive and sustainable communities. Thus, civic dispositions not only contribute to democratic engagement but also serve as affective and ethical foundations for advancing the values and practices of Education for Sustainable Development (ESD) and fostering a more just, equitable, and sustainable future.

**TABLE 3:** Dispositions dimension of civic competencies coding scheme (Kim 2023).

Dimension 3: Civic dispositions	
Subcodes	Definition and examples
Empathy (empathetic)	Being able to identify, understand, or empathize with others’ emotional experiences.
	e.g., ***I feel bad*** *for the Sioux*.
	e.g., ***Sadly*** *, when the Dakota Access Pipeline had started, its path was right through the sacred lands and burial grounds of the native people*.
	e.g**., *I do feel for*** *the government because they did feel sorry for not talking about it and breaking the honorable treaty*.
Perspective taking (considerate)	Being cognizant of others’ viewpoints, status, situations, and experiences; considering the effects of a certain action on other people.
	e.g., ***Imagine*** *signing something that is an agreement that should not be broken*.
	e.g., ***The Settlers needed the land*** *for farming and to let America advance, so they still went to that land and pushed the Native people from there too*.
	e.g., ***The government's point of view*** *is reasonable because there are pros but there are also cons*.
Self‐transcendence (prosocial)	Being willing to provide assistance to others; valuing helping actions; believing that promoting others’ well‐being is a shared concern.
	e.g., *And with all the stuff you don't really need* ***you can leave for the people who actually need the supplies***.
	e.g., *Lastly* ***you could help the community be better*** *and try to help and also do new things so they can get to know more people*.
	e.g., ***I would probably take the canned food and donate it to*** *a homeless shelter or food drive*.
Social capital (open‐minded)	Being willing to learn and embrace diversity (different people and cultures); showing a positive attitude toward people from different backgrounds and conditions.
	e.g., *You could help your community members by* ***adding multicultural foods***.
	e.g., *And then I give 50% of it to Kentucky because* ***there's a lot of people that*** *like* ***don't have*** *homes*, ***don't have*** *electricity or water, they could really use some food right now*.
	e.g., *In my opinion, I think they can have like* ***different cultural foods, say like, you're from India*,** *and you just moved to America, you (wouldn't) just want to eat American food, you would want to have like your own food*.
Respect for human dignity (respectful)	Valuing human basic rights; being willing to advocate for the underrepresented or minority groups.
	e.g., *I think this because* ***everybody should be able to have the right foods***.
	e.g., *So then they can have a healthy or, or a good life so* ***that they can continue to live***.
	e.g., *And the second thing I'm addressing is um what I tell would tell the workers I would tell them* ***it's like disrespectful*** *because there's artifacts and burial sites that are important um to the tribe*.
Critical consciousness (critical)	Having an awareness of a problem; identifying specific deficits of a system.
	e.g., ***It was unfair for the people who*** *liked standing rock because they did not agree with the pipeline going through standing rock*.
	e.g., *And I would tell them that because well*, ***what they're doing right behind me is unkind, unfair, and unnecessary***.
	e.g., *But you can break one when it is a bad treaty cause* ***you can not say okay when it is a bad*** *treaty and go do it*.
Responsibility (Responsible)	Thinking about required actions or desired ways of behaving as citizens; assuming one's personal, political, and economic responsibilities as a citizen; participating in civic affairs in an informed, thoughtful, and effective manner.
	e.g., *Also*, ***I will*** *give Cheryl beans because* ***it will help the environment*** *too*.
	e.g., ***It could damage some things*** *if they go to the water and leaks, it would be* ***bad because um habitats would die*** *that that was in the uh oil and I would tell them that (they should go back to the paths*.
	e.g., *A different option is canceling the oil mining and* ***using a natural resource*** *like solar energy*.
N/A	Does not involve any identified civic dispositions.
	e.g., *I think it is not okay to break any treaty*.
	e.g., *And I have two questions to answer*.
	e.g., *I agree with your response*.

## 4 Results

### 4.1 Descriptive Analysis

The means and standard deviations of focal variables are presented in Tables 4 and 5, and self‐reported focal variables are presented in Table 4. The variables fell under acceptable skewness (<|2|) and kurtosis (<|7|) ranges (Bryne 2010; Hair et al. 2010) and were normally distributed. Descriptive statistics for civic competencies derived from the civic competencies coding scheme are presented in Table 5.

**TABLE 4:** Descriptive statistics of self‐report measures

		Mean (SD)		Skewness	Kurtosis	Range (Min–Max)	
T1 Moral self‐concept		4.14	(0.60)	−0.29	−0.53	2.67	5.00
T1 Civic purpose	Intention	4.01	(0.64)	−0.95	1.80	1.20	5.00
	Motive	4.29	(0.48)	−0.41	−0.30	3.00	5.00
	Action	2.39	(0.75)	0.29	−0.72	1.00	4.00
T2 Moral self‐concept		4.22	(0.66)	−0.62	−0.34	2.17	5.00
T2 Civic Purpose	Intention	4.02	(0.72)	−0.88	1.12	1.20	5.00
	Motive	4.35	(0.54)	−0.93	2.18	1.80	5.00
	Action	2.58	(0.74)	0.07	−0.74	1.00	4.00

*Note*: The values are composite scores based on 5 Likert scales except civic action (4 Likert scale).

**TABLE 5:** Descriptive statistics of civic competencies

	Mean (SD)	Skewness	Kurtosis	Range (Min–Max)	
Civic knowledge	2.02 (1.95)	2.34	6.79	0	11
Civic skills	6.59 (4.00)	1.28	1.89	0	21.5
Civic dispositions	1.41 (1.21)	1.35	2.11	0	6
Total length	7.56 (4.83)	1.45	2.15	1	27

*Note*: Civic knowledge, skills, and dispositions represent the average number of sentences containing valid civic knowledge, skills, and dispositions, respectively. Total length represents the average number of sentences that students generated in each task under a specific modality (essay, Flip, discussion board). For instance, on average, a student generated 7.56 sentences per task under a given modality, and 2.02 sentences contained valid civic knowledge. Range refers to the minimum and maximum average competence levels observed for each student across the three modalities.

Table 6 shows each civic competency subcode's means and standard deviations. A student, on average, generated 29.97 sentences in total across three modalities: individual essays, Flip (self‐recording), and online discussion boards over two units. Each sentence was coded for three dimensions of civic competencies: knowledge, skills, and dispositions. Students generated more NA (Not Applicable) codes for knowledge (*M* = 21.82, *SD* = 15.14) and dispositions (*M* = 24.43, *SD* = 18.28) than for skills (*M* = 3.71, *SD* = 5.36).; 87.62% of sentences received valid civic skills whereas only 18.49%–27.19% of sentences involved valid subcodes in civic dispositions and knowledge, respectively. Social issues (*M* = 2.31, *SD* = 2.66) and economy (*M* = 2.32, *SD* = 3.18) were the most dominant codes for civic knowledge. For civic skills, elaboration skill was most dominant (*M* = 8.50, *SD* = 7.96), followed by positioning (*M* = 5.87, *SD* = 4.63) and justification (*M* = 5.16, *SD* = 3.74). For civic disposition, social perspective taking (*M* = 2.24, *SD* = 2.49) stood out.

**TABLE 6:** Means and standard deviations of subcodes of civic competencies.

	%	Mean	SD	Range	
				Min	Max
Civic knowledge					
Civil society	3.04	0.91	2.00	0	15
Economy	7.74	2.32	3.18	0	19
Knowledge about nutrition	1.74	0.52	1.47	0	9
Knowledge about geography	0.17	0.05	0.25	0	2
International affairs	0.60	0.18	0.49	0	2
Political and government system	2.37	0.71	1.34	0	9
Social historical event	3.84	1.15	2.29	0	14
Social issues	7.71	2.31	2.66	0	12
NA	72.81	21.82	15.14	1	76
Civic skills					
Positioning	19.59	5.87	4.63	0	21
Justification	17.22	5.16	3.74	0	25
Causal reasoning	4.04	1.21	1.58	0	7
Clarification	1.20	0.36	0.76	0	4
Praise	5.41	1.62	2.44	0	19
Counterargument	2.47	0.74	1.55	0	10
Dis/Agreement	2.54	0.76	1.31	0	7
Elaboration	28.36	8.50	7.96	0	41
Expanding	3.74	1.12	1.37	0	5
Hypothetical reasoning	2.57	0.77	1.13	0	5
Referencing	0.50	0.15	0.40	0	2
NA	12.38	3.71	5.36	0	29
Civic dispositions					
Critical consciousness	1.00	0.30	0.72	0	4
Empathy	1.53	0.46	0.86	0	4
Social perspective taking	7.47	2.24	2.49	0	12
Respect	1.57	0.47	0.83	0	4
Responsibility	3.17	0.95	1.57	0	8
Self‐transcendence	2.84	0.85	1.35	0	7
Social capital	0.90	0.27	0.62	0	3
NA	81.51	24.43	18.28	1	90

*Note*: Students generated a total of 29.97 sentences over two units, including all modalities (essay, Flip, discussion board); this equals the sum of mean sentences within each civic competency dimension. Out of an average of 29.97 sentences, for example, students expressed civic knowledge about the economy in 2.32 sentences across two units and three modalities.

Missing data analysis was conducted to identify patterns of missing. Little's MCAR test using SPSS software indicated that the focal variables and covariates used in the analyses were missing completely at random (*p* = 0.443). Therefore, analyses based on the listwise methods for research question 1 did not bias the results. Analyses using Mplus were conducted using full information maximum likelihood (FIML) strategy for research question 2. The correlations between the aforementioned variables are presented in Table 7. Students’ moral self‐concept at pre‐test and post‐test were moderately correlated (*r* = 0.442) and this pattern of correlation was similar for civic purpose (*r* = 0.442–0.547). The correlations among three indices of civic competencies, knowledge, skills, and dispositions were moderate to high (*r* = 0.590–0.801). These civic competencies were significantly correlated with students’ moral self‐concept at both time points (*r* = 0.190–0.239)

**TABLE 7:** Correlations among moral self‐concept, civic purpose, and civic competencies.

	1	2	3	4	5	6	7
1.T1 Moral self‐concept	1						
2.T1 Civic purpose	**0.616**	1					
3.Civic knowledge	0.202	0.056	1				
4.Civic skills	**0.233**	0.068	**0.801**	1			
5.Civic dispositions	**0.239**	0.042	**0.590**	0.702	1		
6.T2 Moral self‐concept	**0.442**	**0.413**	0.190	**0.216**	0.158	1	
7.T2 Civic purpose	**0.386**	**0.547**	0.091	0.086	0.039	**0.675**	1

*Note*: The bold values indicate statistical significance and the *p* values are noted on the diagonal section. Underlined *p *< 0.05, **Bold** *p* < 0.01.

### 4.2 Research Question 1: The Predictability of Civic Competencies on Civic Purpose

Regression analyses were used to address research question 1. Three models were run, each including civic knowledge, civic skills, and civic dispositions as a predictor with a set of covariates: gender, race, district, grade, and family resource (Table 8). In all cases, female students showed lower civic purpose than male students (*β* = −0.254, *SE* = 0.112, *p* < 0.05 for civic knowledge, *β* = –0.262, *SE* = 0.113, *p* < 0.05 for civic skills, and *β* = –0.259, *SE *= 0.114, *p* < 0.05 for civic disposition). Students’ civic purpose at T1 significantly predicted civic purpose at T2 in all models (*β* = 0.551, *SE* = 0.099, *p* < 0.001 for civic knowledge model, *β* = 0.548, *SE *= 0.099, *p *< 0.001 for civic skills model, and *β* = 0.546, *SE *= 0.099, *p *< 0.001 for civic disposition model). However, none of the civic knowledge, skills, and dispositions predicted civic purpose at T2 after controlling for civic purpose at T1 and covariates.

**TABLE 8:** (RQ1) Predictability of civic competencies on civic purpose.

	T2 Civic purpose					
	*B*	*SE*	*B*	*SE*	*B*	*SE*
	[95% CI]		[95% CI]		[95% CI]	
Gender (Female = 1)	−0.254^*^	0.112	−0.262^*^	0.113	−0.259^*^	0.114
	[−0.474 to −0.034]		[−0.484 to −0.041]		[−0.483 to −0.035]	
Race (White = 1)	−0.057	0.129	−0.058	0.129	−0.051	0.130
	[−0.310 to 0.196]		[−0.311 to 0.196]		[−0.306 to 0.203]	
District (District A = 1)	0.011	0.164	0.025	0.161	0.041	0.158
	[−0.311 to 0.333]		[−0.290 to 0.341]		[−0.269 to 0.350]	
Grade (5th grade = 1)	0.085	0.126	0.062	0.118	0.050	0.117
	[−0.163 to 0.333]		[−0.170 to 0.294]		[−0.180 to 0.280]	
Family resource	0.010	0.039	0.011	0.039	0.009	0.039
	[−0.067 to 0.087]		[−0.066 to 0.087]		[−**0**.068 to 0.085]	
T1 Moral self‐concept	0.097	0.122	0.100	0.122	0.106	0.124
	[−0.141 to 0.335]		[−0.139 to 0.339]		[−0.137 to 0.349]	
T1 Civic purpose	0.551^***^	0.099	0.548^***^	0.099	0.546^***^	0.099
	[0.357 to 0.744]		[0.355 to 0.741]		[0.352 to 0.741]	
Civic knowledge	0.022	0.031				
	[−0.040 to 0.083]					
Civic skills			0.008	0.015		
			[−0.021 to 0.037]			
Civic dispositions					0.010	0.048
					[−0.084 to 0.104]	

*Note*: *Z* scores of civic purpose subscales were calculated and averaged to derive composite scores, accounting for the different Likert‐based subscales. *p<.05, **p<.01, ***p<.001

### 4.3 Research Question 2: The Mediating Role of Moral Self‐Concept Between Civic Competencies and Civic Purpose

We explored the mediation effect of moral self‐concept at T2 in the association between students’ civic competencies (knowledge, skills, and dispositions) and civic purpose at T2. Student demographic backgrounds and civic purpose at T1 were controlled and a bootstrapping test was employed, using 500 samples (Table 9).

**TABLE 9:** (Research Question2) The Mediating role of moral self‐concept between civic competencies and civic purpose.

		*β*	*SE*	CI
Civic Knowledge	Civic knowledge →Moral self‐concept (a)	0.067^*^	0.027	[0.014 to 0.117]
	Moral self‐concept →Civic purpose (b)	0.674^***^	0.112	[0.444 to 0.877]
	Civic knowledge →Civic purpose (c)	−0.016	0.028	[−0.076 to 0.038]
	Civic knowledge →Moral self‐concept →Civic purpose (a→b→c)	0.045^**^	0.017	[0.010 to 0.076]
Civic skills	Civic skills → Moral self‐concept (a)	0.035^**^	0.011	[0.011 to 0.058]
	Moral self‐concept → Civic purpose (b)	0.682^***^	0.112	[0.448 to 0.882]
	Civic skills → Civic purpose (c)	−0.012	0.011	[−0.036 to 0.008]
	Civic skills → Moral self‐concept → Civic purpose (a→b→c)	0.024^**^	0.008	[0.007 to 0.040]
Civic dispositions	Civic dispositions → Moral self‐concept (a)	0.074	0.044	[−0.010 to 0.159]
	Moral self‐concept → Civic purpose (b)	0.706^***^	0.112	[0.472 to 0.897]
	Civic dispositions → Civic purpose (c)	−0.032	0.038	[−0.111 to 0.043]
	Civic dispositions → Moral self‐concept → Civic purpose (a→b→c)	0.053^+^	0.030	[−0.007 to 0.109]

*Note*: (a) Represents the effect of each civic competence on moral self‐concept, (b) represents the effect of moral self‐concept on civic purpose, (c) represents the direct effect of each civic competence on civic purpose, *a→b→c* represents the indirect effect of each civic competence on civic purpose through moral self‐concept. +*p* < .10, *p < .05, **p < .01, ***p < .001

Although the result from research question 1 showed that civic competencies did not predict civic purpose, examining a mediation effect while the direct effect is nonsignificant remains a logical approach since the existence of significant association between X and Y is not a precondition in testing a mediation effect (Hayes 2017). Even though mediation analyses would not guarantee causal inferences (Vo et al. 2020), the time points of measures, where civic competencies (X) were captured during students’ participation in DCL prior to the assessment of moral self‐concept (M) and civic purpose (Y), enabled us to interpret that students’ civic competencies serve as a precursor in informing moral self‐concept, which in turn mediates the relation between civic competencies and civic purpose (see Gunzler et al. 2013).

Mediation analyses using Mplus showed acceptable model fits: civic knowledge (CFI = 1.000, TLI = 1.000, RMSEA = 0.000, SRMR = 0.040), civic skills (CFI = 1.000, TLI = 1.000, RMSEA = 0.000, SRMR = 0.042), and civic dispositions (CFI = 0.962, TLI = 0.905, RMSEA = 0.079, SRMR = 0.056). The results are presented in Table 9 based on the standardized coefficients. There was a significant mediation effect of moral self‐concept (T2) in the relation between civic knowledge and civic purpose (T2) (*β* = 0.045, *SE* = 0.017, *p *< 0.01, 95% bootstrap CI [0.010, 0.076]) as well as civic skills and civic purpose (T2) (*β* = 0.024, *SE* = 0.008, *p *< 0.01, 95% bootstrap CI [0.007, 0.040]). However, this trend was marginally significant in the association between civic dispositions and civic purpose (*β* = 0.053, *SE* = 0.030, *p *< 0.10, 95% bootstrap CI [–0.007, 0.109]).

## 5 Discussion

This study contributes to emerging scholarship at the intersection of civic education and sustainability by highlighting moral self‐concept as a critical developmental construct that links civic competencies to a deeper, more enduring sense of civic purpose. While civic competencies provide the requisite knowledge and skills for civic engagement (Ballard and Ozer 2016), our findings suggest they are not sufficient to sustain a long‐term orientation toward social and ecological well‐being, a key aim of Education for Sustainable Development (ESD) as articulated in SDG 4.7. Instead, it is the internalization of civic and moral values—reflected in early adolescents’ developing moral self‐concept—that transforms competencies into meaningful, sustained engagement.

DCL provided a rich learning and social context where students can demonstrate their civic competencies by generating and articulating civic knowledge and showing intellectual and social skills motivated by different civic dispositions. Based on the descriptive findings, it is noteworthy that the social perspective taking disposition appeared most frequently in students’ video essays, discussion posts, and reflective essays. One possible explanation is that participants in this study, ranging from 8 to 11 years old, were in the active developmental period experiencing decentralization, where they gradually shift away from egocentrism by expanding their understanding of others’ perspectives (Hoffman 2008; Spinrad and Eisenberg 2014).

Another noteworthy descriptive finding is that students demonstrated a relatively wider range in their civic skills than in their civic knowledge or dispositions. This can be partially explained by the study context, where positive social norms of group discussions actively encourage students to participate in dialogue. Because simple expressions, such as dis/agreeing to others’ ideas respectfully or praising groupmates’ contributions, were coded as valid civic skills, students could readily demonstrate these skills as part of adhering to positive social norms. This likely contributed to higher means and a wider range for skills compared to knowledge or dispositions. With that, civic skills that stood out compared to knowledge or dispositions in this study may be interpreted with the sociocultural perspective that participation by itself serves as a process of appropriation (Rogoff 1995; Vygotsky 1978).

Surprisingly, none of the civic knowledge, skills, or dispositions demonstrated by students during the DCL significantly predicted civic purpose at post‐test, once background characteristics, moral self‐concept, and baseline civic purpose were accounted for. While this finding challenges dominant assumptions in civic education scholarship, which often emphasize civic competencies as foundational for youth civic development (e.g., Diković and Zečević 2020; Ten Dam et al. 2011), it also suggests these competencies may still contribute to mature citizenry indirectly through a fostered moral self‐concept.

Our findings on the mediating role of moral self‐concept further suggest that the development of a moral self is foundational to cultivating civic purpose in early adolescents. Early adolescents with a strong moral self‐concept are more likely to experience empathy, intrinsic motivation, and a sense of accountability (e.g., Castro‐Sánchez et al. 2019)—all essential for engaging with complex sustainability issues such as inequality, environmental justice, and community resilience (SDG 16). Developmentally, there has been mixed findings and arguments about whether moral self‐concept in early adolescence is reliable or not (see Kingsford et al. 2018), and whether the integration of morality into oneself is available to this age or not (see Krettenauer and Hertz 2015). However, the findings speak to the plausibility of the self‐congruence principle (Blasi 1983), working similarly in young students as young as early adolescents; high moral self‐concept, assessed as the centrality and perceived importance of moral values such as being just, helpful, and sympathetic, serves as a bridge connecting their competencies (e.g., civic knowledge) and commitment (e.g., civic purpose). Even if our findings can only speak to the implications specifically for early adolescents, previous empirical studies on adolescents in general seem to support such an approach of cultivating moral self‐concept as a way to nurture mature citizens for sustainable development; adolescents who perceive themselves as moral are more likely to participate in civic activities such as volunteering in both middle adolescence (*M*_age_ = 15.5; Hart and Fegley 1995) and late adolescence (*M*_age_ = 17.4; Porter 2013), although findings are not always consistent (e.g., Pratt et al. 2003).

It is also possible that students’ engagement in the socially‐ and morally‐complex, open‐ended group projects in DCL required them to reflect on and examine their own value systems. This process encourages them to examine their own belief systems, thereby cultivating relevant moral values (García‐Moriyón et al. 2020). For instance, student‐generated responses on the discussion board, self‐recording software, and online individual essays reflected such mechanisms. Students’ remarks represented the emerging moral values such as being “considerate” (e.g., *“Imagine signing something that is an agreement that should not be broken.”*), “sympathetic” (*“I feel bad for the Sioux.”*), “fair/just” (e.g., *“It was unfair for the people who liked standing rock because they did not agree with the pipeline going through standing rock.”*), and “responsible” *(“I will give Cheryl beans because it will help the environment too”*), to name a few. Thus, DCL seems to have provided valid learning contexts where students, as communicative beings (Shor and Freire 1987), co‐create knowledge through repeated and well‐designed reflections, while engaging with the world and recreating their understanding of society, dialectically (Robert 1998). In line with this, DCL's curricular features with situated conversations involving value judgments might have expanded and promoted “self‐transcendence,” leading to civic purpose; discussion prompts challenge students to think about societal issues, responsibilities in different roles and contexts, basically asking them to reason beyond personal concerns. This process helps students recognize person‐community relations and one's roles in the social world (see Houser 2007), tapping into the notion of praxis, that is, reflection and action upon the world (Freire 1970).

Taken together, the findings underscore the need to move beyond technical or procedural civic education. Preparing youth for sustainability challenges requires engaging the moral, emotional, and existential dimensions of development as an integrated whole. Cultivating moral self‐concept during early adolescence may be a developmentally strategic and equity‐driven approach to fostering purposeful, sustainability‐minded citizens.

### 5.1 Significance and Contribution

This study contributes to developmental science by clarifying the mechanisms through which civic development unfolds within the framework of Education for Sustainable Development (ESD), particularly through the pedagogical model of Digital Civic Learning (DCL). The findings highlight the central role of moral self‐concept in bridging civic competencies and civic purpose, especially during early adolescence—a developmental window characterized by heightened self‐reflection and identity formation. This underscores the importance of educational interventions that attend not only to what children learn about, but also to who they are becoming as ethical, responsible, and future‐oriented individuals.

Specifically, this study extends the existing knowledge on civic competencies and sustainability education by providing detailed snapshots of how early adolescents actually participate, engage in, and demonstrate their competencies in their daily learning environments within sustainability education settings, particularly using digital tools and in digital spaces. In order to realistically capture these civic competencies in a digital learning setting, we employed a performance‐based assessment approach to reveal students’ civic knowledge, skills, and dispositions as demonstrated through their online discussions, individual writing, and self‐recorded videos over the DCL curriculum, tapping into digital citizenship. Echoing other researchers that digital citizenship must be understood as wider than traditional forms of civic engagement and action (Choi et al. 2017; Kahne et al. 2013), civic competencies, demonstrated in class with a digital civic curriculum, competently formulates digital community among students (Hollandsworth et al. 2011). As much as classroom‐based, character education‐aligned digital citizenship learning for K‐12 education is needed, the study advances the knowledge of sustainability education and civic competencies by revealing where students in this age group stand in terms of their civic knowledge, skills, and dispositions, while further providing empirical support on the role of moral self‐concept as a developmental asset linking such civic competencies and civic purpose in early adolescence.

These forms of student‐generated data, embedded within the flow of authentic learning experiences, offer distinct advantages from an ESD perspective because civic participation within the classroom can mirror civic life in broader social contexts—providing a living laboratory for cultivating the competencies that underpin sustainable and participatory societies (Kirlin 2003). Though such an approach of authentic, organic performance‐based assessments reveals how students’ civic competencies would unfold in real‐life settings, it is inherently influenced by a range of individual and contextual variables: academic motivation (e.g., willingness to engage in discourse), peer dynamics (e.g., feelings of safety in expressing views), content comprehension, and technological self‐efficacy. Rather than viewing these as confounding variables, ESD frames them as integral to the development of civic agency. Thus, the measures we developed with the new coding scheme may not fully isolate individuals’ personal and surrounding factors, they offer greater ecological validity than decontextualized, self‐reported perceptions. From an ESD lens, civic competencies—such as critical thinking, collaboration, intercultural understanding, and ethical reflection—are not fixed attributes but context‐sensitive capabilities that evolve through participation in meaningful, dialogic, and socially embedded learning. Our exploratory approach—using authentic learning performances to assess civic competencies—provides an important contribution to the field, offering new insights into the complex developmental pathways that underlie sustainability‐oriented citizenship.

### 5.2 Study Limitation and Future Research

Because the study framed civic purpose as a developmentally desirable outcome in early adolescence, civic purpose was assessed only once after the DCL curriculum, without follow‐up measurements. This design limits the ability to determine whether students’ civic purpose is sustained over time. Conceptually, civically purposeful youth would maintain both their desire and engagement in civic activities over time, yet little is known about whether, and for how long, civic purpose changes or is maintained in response to educational inputs such as curricula. Future research should therefore incorporate follow‐up assessments to clarify the developmental course and stability of civic purpose in early adolescents.

Additionally, since moral self‐concept, positioned as a mediator, was measured concurrently with civic purpose, the findings cannot provide evidence for causal inference, which would require civic purpose to be assessed at a later time point. Therefore, the interpretation of moral self‐concept as mediating the relationship between civic competencies and civic purpose should be situated within existing theoretical frameworks, such as moral identity theory and the self‐congruence principle.

Another promising direction for future research would be to apply a microgenetic approach that examines the processual development of civic competencies over time. This would allow researchers to zoom in and trace how students construct, revise, and internalize civic ideas and values—yielding deeper insights into the developmental trajectories of civic competencies and related learning outcomes.

## 6 Conclusion

Aligned with the aims of ESD and the targets of SDG 4.7, *Quality Education*, the results emphasize the value of holistic educational approaches that integrate cognitive, emotional, and moral dimensions of learning. The DCL curriculum exemplifies how embedding complex, real‐world civic dilemmas into students’ learning—through both digital and in‐person modalities—can provide rich opportunities for critical thinking, moral reflection, and the construction of civic purpose.

Educators who align their instruction with ESD principles—by incorporating critical inquiry into sustainability challenges, promoting inclusive and participatory dialogue, and connecting civic content to students’ lived experiences—can support students in developing not only the capabilities but also the intrinsic motivation to act on behalf of the common good. Such approaches advance the goals of ESD by preparing students to engage with complexity, recognize interconnectedness, and contribute meaningfully to building a more positive and sustainable future.

Educators need to actively employ participatory, technology‐based immersive, experiential learning models such as DCL in their classrooms. The emphasis that such curricula place on ethically charged dilemmas, values‐based reflection, and multi‐stakeholder perspectives align directly with the aims of ESD, which promotes not only cognitive skills but also the emotional and ethical dimensions of sustainable development.

## Ethics Statement

All procedures performed in the study involving human participants were in accordance with the ethical standards of the institutional research committee.

## Conflicts of Interest

The authors declare that they have no conflict of interest.

## Data Availability

The data used in this study are not publicly available due to the IRB regulations, but may be made available from the corresponding author on reasonable request with appropriate ethical approvals.

## Endnotes

1 Ms. Haaland is Native American politician who appears in the reading materials of the DCL, Unit 3: History.
